# Repurposable Drugs for Immunotherapy and Strategies to Find Candidate Drugs

**DOI:** 10.3390/pharmaceutics15092190

**Published:** 2023-08-24

**Authors:** Norihiro Sakai, Kenya Kamimura, Shuji Terai

**Affiliations:** 1 Division of Gastroenterology and Hepatology, Graduate School of Medical and Dental Sciences, Niigata University, 1-757, Aasahimachi-Dori, Chuo-Ku, Niigata 951-8510, Japan; nsakai@med.niigata-u.ac.jp (N.S.); terais@med.niigata-u.ac.jp (S.T.); 2 Department of General Medicine, Niigata University School of Medicine, 1-757, Aasahimachi-Dori, Chuo-Ku, Niigata 951-8510, Japan

**Keywords:** repurposing drug, genetic alterations, immunotherapy

## Abstract

Conventional drug discovery involves significant steps, time, and expenses; therefore, novel methods for drug discovery remain unmet, particularly for patients with intractable diseases. For this purpose, the drug repurposing method has been recently used to search for new therapeutic agents. Repurposed drugs are mostly previously approved drugs, which were carefully tested for their efficacy for other diseases and had their safety for the human body confirmed following careful pre-clinical trials, clinical trials, and post-marketing surveillance. Therefore, using these approved drugs for other diseases that cannot be treated using conventional therapeutic methods could save time and economic costs for testing their clinical applicability. In this review, we have summarized the methods for identifying repurposable drugs focusing on immunotherapy.

## 1. Introduction

The development of novel therapeutic methods for intractable diseases is urgently needed. Among the various methodologies examined, immunotherapy is the therapeutic option which suppresses or enhances the patients’ immune system [1]. Therapy to suppress the immune system has been used for autoimmune diseases and allergy, and to prevent rejection after organ transplantation. Therapy to enhance the system has recently been used for cancer immunotherapy [2,3]. However, the cost and time to develop new drugs for immunotherapy is enormous, and it is possible that the momentum of new drug development in this area will decrease in the future. Therefore, among several strategies and studies to fulfill this unmet need, drug repurposing is one of the promising methods [4,5,6]. The major advantage of this method is that drugs are approved for their safety based on trials; therefore, it could save costs and time for drug development. To date, the method has been focused on various fields, including infectious diseases [7,8], cardiovascular diseases [9], neurological diseases [10], and rheumatic autoimmune inflammatory diseases (RAIDs) [11]. In particular, limited understanding of the etiology of RAIDs, an autoimmune disease, makes drug repurposing for RAIDs difficult. Nevertheless, the past century has seen the discovery of a variety of repurposes. Early repurposing of agents was based on serendipity or recognition of disease similarities. Serendipitously effective clinical cases include penicillamine for rheumatoid arthritis (originally developed for Wilson’s disease) and hydroxychloroquine for systemic lupus erythematosus (originally developed for malaria), which were both useful as effective immunotherapy for the refractory RAIDs [11]. Recently, this method has attracted attention for the development of therapeutic strategies for severe acute respiratory syndrome coronavirus 2, which caused the coronavirus disease 2019 (COVID-19) pandemic for which we had no basic research information or experience in treating it [7,12]. While utilizing this method, we have extracted potential candidate drugs to be used for improving case symptoms and realized that “drug repurposing” is a reasonable and effective method for searching for drugs that can be used for various diseases for which effective therapeutic strategies have not been established.

Moreover, we have recognized that these repurposable drugs have been picked up unexpectedly and by chance in the past years. Recently, the use of genome-wide information has been reported for the usefulness of the discovery [13]. In this review, we have summarized strategies for finding candidate drugs, including genetic alterations, target molecules, and in-silico-based searches to identify repurposable drugs, focusing on immunotherapy. This information will contribute to the development of new drugs for intractable diseases economically and in a timely manner.

## 2. Drug Repurposing

Drug repurposing is also known as drug repositioning. The major advantages of the process are reducing the time and costs required to develop the novel drugs. This is due to the fact that these approved drugs are tested in step-by-step preclinical studies of Phase 1, 2, 3, and 4, and safety and efficacy data are collected. In addition, the safety profiles of the developed drugs are continuously collected in the real-world setting after being put on the market. Therefore, compared to the traditional novel drug developmental procedure, drug repurposing is a useful process to discover new drugs, especially for rare diseases and those which cannot be treated by conventional drugs. Although the original efficacy of the drug may have side effects, and great care must be taken to eliminate such side effects, drug repurposing is considered a useful method of therapeutic development for rare or refractory diseases. To identify the candidates of the repurposable drug, various experimental and data-driven approaches have been utilized. In this review, we have summarized the strategies to find candidate drugs focusing on the immunotherapy. Firstly, we discussed the repurposable drugs for immunotherapy (Table 1). These include rapamycin, metformin, pentostatin, MSDC-0160, aspirin, celecoxib, niclosamide, denosumab, riluzole, and digoxin. Then, we summarized the topic of the discovery of repurposing drugs on the basis of gene expression analyses (Table 2). The drugs involved in this section include vorinostat, mocetinostat, menadione, and letrozole.

## 3. Repurposing Drugs for Immunotherapy

**Figure 1:**
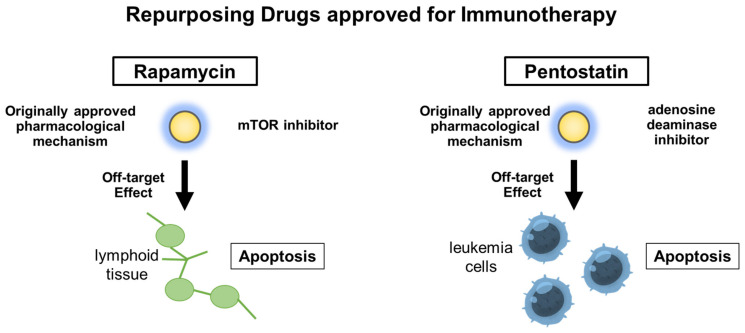
Mechanism of the repurposed drugs approved for immunotherapy.

Before summarizing the strategies for finding candidate drugs, we first summarized repurposable drugs identified to date, including unexpectedly discovered drugs (Table 1). In addition, to demonstrate the mechanism of the repurposed drugs, Figure 1 shows the representative drugs of rapamycin and pentostatin.

### 3.1. Rapamycin

Rapamycin is originally used in preventing organ transplant rejection; however, a new indication is the treatment of autoimmune lymphoproliferative syndrome (ALPS). In one case series, rapamycin was administered to four pediatric patients with ALPS and subsequently improved cytopenias and reduced lymphoproliferation [14]. The mechanism is believed to be the antiproliferative properties of mammalian target of rapamycin (mTOR) inhibitors; it is used as a secondary treatment for ALPS [50]. Furthermore, a decrease in lymph node and spleen size was observed in the murine ALPS model following rapamycin treatment [15]. Using its lymphoproliferative inhibitory effect, it is also starting to be used in the treatment of lymphatic malformations in children [51].

### 3.2. Metoformin

Metformin is a drug that has long been used for treating type 2 diabetes. Based on the big data of cases that used metformin for diabetes treatment, a new indication has been observed for advanced prostate cancer [16]. For example, a Canadian cohort study including 3837 patients reported that the increased cumulative duration of metformin was associated with reductions in both all-cause and prostate-cancer-specific mortality in patients with prostate cancer with diabetes mellitus [52,52]. In vitro experiments have shown that metformin inhibits the mammalian target of rapamycin complex 1 (mTORC1) and upregulates AMP-activated protein kinase [17,18]. These results suggest that metformin could be used for prostate cancer in patients without diabetes. In addition, clinical big data have reported metformin and various types of cancer inhibition [53]; the master pathway of metformin anticancer activity is thought to be the activation of AMPK/mTOR pathway triggered by inhibition of complex I in the mitochondrial respiratory chain [54,55].

### 3.3. Pentostatin

Pentostatin has been used for treating T-cell–related leukemia; however, its immunosuppressive effect has also been observed to be effective for the treatment of B-cell–related hairy cell leukemia [19]. The efficacy of pentostatin was reported in a randomized comparison including 313 patients using pentostatin versus those using interferon alfa-2a in previously untreated patients with hairy cell leukemia [56]; pentostatin has been recommended as a first-line therapy for hairy cell leukemia [57]. The effect was mainly caused by the adenosine deaminase inhibition inducing the deoxyadenosine triphosphate accumulation which caused DNA strand breaks in leukemia cells followed by the activation of apoptotic pathway via p53 and cytochrome c release from mitochondria [20]. Although there has been enormous progress in understanding the biology of hairy cell leukemia over the past few decades, pentostatin is still one of the drugs used as an initial therapy [58].

### 3.4. MSDC-0160

MSDC-0160 has been used for type 2 diabetes; however, it is now used for Parkinson’s disease (PD) as a new indication. It has been reported that PD and diabetes mellitus share similar pathophysiological characteristics [21] as well as genetic and environmental factors and that individuals with diabetes mellitus appear to have a remarkably higher incidence of PD than age-matched non-diabetic individuals. Furthermore, it is reported that the use of the antidiabetic agent, glitazone, is associated with a decreased risk of PD incidence in patients with diabetes [59]. The mechanism of MSDC-0160 for PD is believed to be the reduction in the inflammation level and a decrease in nerve death; moreover, it acts as a carrier of pyruvate to the mitochondria [22]. A clinical trial was started with the results of the basic research, and the Phase II study has just been completed [60]. In the Phase II study, MSDC-0160 significantly reduced blood glucose and HbA1c levels compared with the placebo group; however, this has not yet been reported for PD [61].

### 3.5. Aspirin

Aspirin is a prototype non-steroidal anti-inflammatory drug for treating various pain and inflammatory disorders. It is also widely used for cardiac and cerebrovascular diseases due to its antiplatelet action [62,63]. A new indication of aspirin aims at manipulating anticancer immunity on its efficacy as an antiplatelet drug. Recently, it has been reported that platelet activation represents a mechanism of immune evasion that mediates CD8+ T-cell function suppression within the tumor microenvironment, and the efficacy of aspirin as an antiplatelet drug enhanced responsiveness to programmed death receptor-1 (PD-1) blockade [23]. Furthermore, aspirin supports the elimination of tumor cell debris by activating macrophages and blocking pro-inflammatory cytokine secretion from decaying cancer cells [24]. A clinical trial for the treatment of cervical and uterine cancer showed efficacy of aspirin combined with pembrolizumab and radiotherapy [24,25]. A nationwide study in US revealed possible prognostic benefit in aspirin users with hepatocellular carcinoma [64]. A total of 224,735 patients were included in the study. Of them, 18,835 (8.4%) were long-time aspirin users. Patients in the aspirin group had decreased rates of hepatic decompensation and lower incidence of sepsis, shock, acute kidney injury (AKI), intensive care unit (ICU) admission, and in-hospital mortality.

### 3.6. Celecoxib

Celecoxib was originally used for osteoarthritis; however, a new indication has been approved for reducing the risk of additional polyp formation in colon cancer [65]. Celecoxib administration reduced the incidence of colorectal cancer in the general population [66]. However, a randomized clinical trial called the CALGB/SWOG 80,702 (Alliance) showed that among patients with stage III colon cancer, the addition of celecoxib for 3 years, compared with placebo, to standard adjuvant chemotherapy did not significantly improve disease-free survival [26]. In a review of celecoxib as an add-on to standard chemotherapy, 13 randomized controlled trials (RCTs) of breast, non-small cell lung cancer (NSCLC), gastric, bladder, ovarian, colon, and prostate cancer were examined, and none showed clear overall-survival (OS) or progression-free survival (PFS) improvement, suggesting a potential for increased hematologic toxicity [67]. However, the synergic effect of pharmacological blockade of COX-2 (celecoxib or aspirin) with immune checkpoint blockade was revealed in preclinical models [68]. A recent retrospective study showed that use of NSAIDs may improve OS in patients receiving immune checkpoint blockade therapy [69]. Its effects are believed to be because of Cox-2 inhibition, NF-kB activity inhibition, and interference with the binding of PPARγ to DNA [26,27].

### 3.7. Niclosamide

Niclosamide was initially approved for anti-helminthic treatment. Recently, several research groups reported its potential as an anticancer drug. It interferes with tumor progression and metastasis via S100A4 inhibition. It inhibits tumor growth and invasion in cisplatin-resistant human epidermal growth factor receptor-2 (HER2)-positive breast cancer by reversing EMT and inhibiting stemness and invasion [28]. Moreover, niclosamide blocks metastasis in hepatocellular carcinoma cell lines by downregulating twist-mediated CD10 expression [29]. The mechanisms of the effect of niclosamide for malignancies are believed to be because of its interference with various cancer pathways, including PI3K/Akt, Wnt/β-catenin, Jak/STAT, and NF-kB signaling [30]. In vitro, niclosamide in combination with PD-1/programmed cell death ligand 1 (PD-L1) antibody has a synergistic anti-tumor effect in non-small cell lung cancer models through decreasing PD-L1 expression and promoting cytotoxic T-cell activity [31]. A phase II trial was previously conducted to investigate the safety and efficacy of orally applied niclosamide in patients with colorectal cancer metastases [32]. In addition, a review reported that niclosamide could be a drug repurposed for the neurotherapy of autism by targeting mitochondrial dysfunction [70]. This hypothesis is based on the expectation that niclosamide will act on the extracellular signal-regulated kinase (ERK)/MAPK pathway and improve mitochondrial dysfunction, which is thought to be a factor in autism spectrum disorders [71]. A study in osteosarcoma cell lines revealed that niclosamide can prevent the phosphorylation of ERK1/2 [72].

### 3.8. Denosumab

Denosumab is an inhibitor of the receptor activator of nuclear factor kappa B (RANK) ligand (RANKL) and was originally approved for therapy of skeletal-related events in patients with advanced conditions including solid tumors and multiple myeloma. The RANK/RANKL system regulates bone homeostasis through bone remodeling and osteoclast function. On the other hand, the emerging role of the RANKL/RANK signaling axis in mammary gland development, cancer metastasis, hormone-derived breast cancer development, and thermal regulation has been investigated [33]. Following improved understanding of the role of RANK/RANKL in cancer biology, denosumab has already been repurposed as a treatment for giant cell tumor of bone. Furthermore, some phase II clinical trials evaluate a combination of denosumab and immune checkpoint inhibitors (ICIs) for the treatment of metastatic melanoma [34].

### 3.9. Riluzole

Riluzole is an inhibitor of glutamate release that is FDA-approved for the treatment of Amyotrophic Lateral Sclerosis (ALS). It reduced tumor cell proliferation in vitro and tumorigenesis in vivo [35]. Based on these observations, a 12-patient, pilot phase 0 trial of riluzole treatment was conducted in patients with stage III and IV melanoma prior to surgical resection [36], and associated decrease in metabolic activity on post-treatment PET-CT scans. However, a subsequent phase II study of riluzole in patients with stage III unresectable or stage IV melanoma showed no responses in the first 13 patient and trial was terminated [37]. Recently, it has become known that overexpression of metabotropic receptor 1 (GRM1) has been implicated in the pathogenesis of multiple cancers. Riluzole, an inhibitor of glutamate release, is once again in the spotlight and showed synergistic anti-tumor activity with the multi-kinase inhibitor sorafenib in preclinical models [38]. Based on these results, a phase I trial of riluzole and sorafenib in patients with advanced solid tumors including melanoma, colorectal cancer, cervical cancer, lung cancer, pancreatic cancer, ovarian cancer, urothelial tumor, and sarcoma was conducted [39].

### 3.10. Digoxin

Digoxin, a drug long used for treating congestive heart failure and arrhythmias, is attracting attention for its new indication for various disease such as steatohepatitis [73], rheumatoid arthritis [74], and cancer [40]. A systemic review of ten clinical studies with a total of 108,444 participants (15,835 individuals were digoxin users) reported a preventive effect of digoxin usage for the risk of prostate cancer in men [41]. The method used for discovering its anticancer effects was a screen test of 3120 FDA-approved drugs using comprehensive gene analysis against cell line Hep3B-c1. Further, the study showed that digoxin acts as a hypoxia-inducible factor1 (HIF-1) inhibitor, and its anticancer effect was confirmed in in vivo xenograft models [42]. Suppressing the HIF-1 pathway in prostate cancer cells is reported to lead to anti-tumor effects [75]. This is an example of the strategy for finding candidate drugs for repurposing utilizing the comprehensive analysis of gene expressions, and we describe this strategy in the next paragraph.

## 4. Discovery of Repurposing Drugs on the Basis of Gene Expression Analyses

Drugs discovered on the basis of gene expression analyses are summarized in Table 2.

### 4.1. Vorinostat

Vorinostat (suberoylanilide hydroxamic acid: SAHA) is a histone deacetylase inhibitor originally used for cutaneous T-cell lymphoma; however, based on gene expression data for human HER2-positive breast cancer, a new indication for HER2-positive breast cancer was discovered. First, gene expression data for breast invasive carcinoma were retrieved from The Cancer Genome Atlas (TCGA) database. The dataset contained the genes involved in four different subtypes including Luminal A, Luminal B, HER2 and Triple negative, and also four different stages of breast cancer including I, II, III, IV, as well as normal specimens [43]. HER2 subtype was extracted from a total of 591 samples (526 cancer cases, 65 normal cases, and 17,814 genes). Using Linear Models for Microarray Data (LIMMA) package, the top 100 differentially expressed genes (DEGs) were found. These genes were used as an entry for the library of integrated network-based cellular signatures (LINCS) L1000CDS2 software which suggested 24 repurposed drugs, including vorinostat [44]. The mechanism is determined using breast cancer BT-474 and SKBR-3 cells indicating that vorinostat causes heat-shock protein 90 (HSP90) acetylation, which leads to the dissociation of HSP90 from HER2, thereby resulting in polyubiquitin chain synthesis and HER2 degradation [76].

### 4.2. Mocetinostat

Mocetinostat (MGCS0103) has been known as an orally available and small-molecule class I histone deacetylase (HDAC) inhibitor that induces tumor necrosis factor α expression and secretion [77]. It was originally used for classical Hodgkin’s lymphoma and induces apoptosis and simultaneously increases NF-kB and PD-L1 expression in classical Hodgkin’s lymphoma [45]. However, a new indication for HER2–positive breast cancer was discovered from comprehensive genetic analysis data [46] for human HER2-positive breast cancer. It is demonstrated that co-treatment with bromodomain extra-C terminal domain inhibitors and HDAC inhibitors, including mocetinostat, decreases breast cancer cell viability by the induction of ubiquitin-specific protease 17 (USP17) [78]. The highest degree in the drug-gene network is observed with HDAC1 which is a target gene for the repurposed drugs vorinostat and mocetinostat [44].

### 4.3. Menadione

Menadione has been previously used for vitamin K supplementation; however, currently it is not used for vitamin K supplementation owing to its toxicity. Based on gene expression data, its new indication for luminal B pattern breast cancer has been discovered, and it is believed to inhibit breast cancer cell growth by acting on F10 and EGFR genes [47]. Furthermore, menadione/ascorbate (M/A; also named Apatone^®^) attract attention with their ability to kill cancer cells without affecting the viability of normal cells, and the effects of M/A for breast cancer cells (MCF7), colon cancer cells (Colon26), and leukemic lymphocytes (Jurkat) were examined. M/A exhibited highly specific and synergistic suppression on cancer cell growth but without adversely affecting the viability of normal cells at pharmacologically attainable concentrations [48].

### 4.4. Letrozole

Letrozole has been used for breast cancer; however, a new indication for liver fibrosis has been discovered, focusing on comprehensive gene alternations in chimeric mice models with humanized hepatocytes. After 36 repositionable drugs were administrated to chimeric mice, microarray on human genes was performed and indicated that gene expression analysis was an appropriate method for screening for medicines that were effective for liver fibrosis and that LET inhibited YAP, CTGF, and TGF-β expressions and activated CYP26A1 expressions in humanized hepatocytes in chimeric mice; moreover, it ameliorated liver fibrosis in two liver fibrosis mouse models [49].

## 5. Discussion

**Figure 2:**
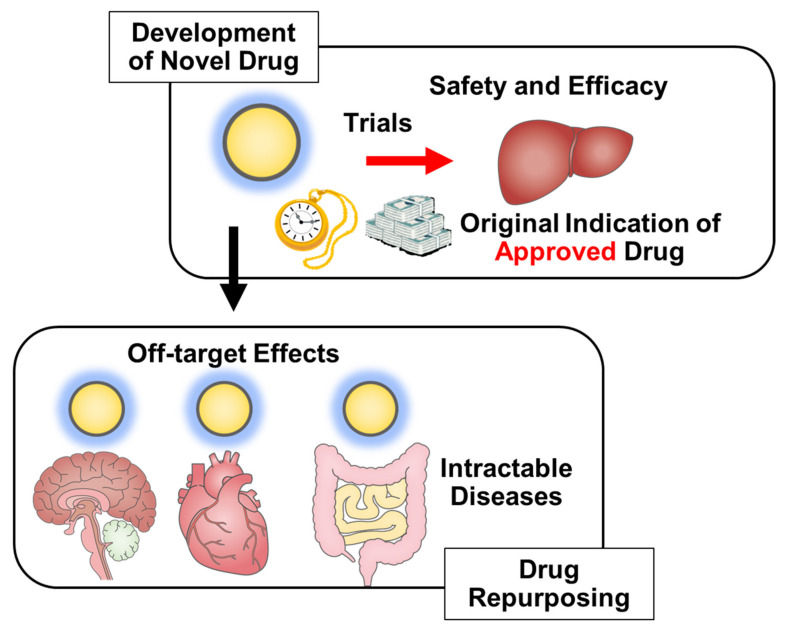
Advantages of Drug Repurposing.

To date, the development of a new drug requires 13–15 years and between USD 2 billion and USD 3 billion of investment on average [79]. Although repurposing drugs have frequently been discovered by chance, the ability to use originally approved drugs for new treatments has advantages regarding safety, cost savings, and early adaptation for treating new diseases. Additionally, new indications can be discovered by analyzing large amounts of data from originally used indications; in such cases, data are real-world clinical data in actual humans, with more validity expected. Although we have introduced a few repurposing drugs, several others are being used in the repurposing drug approach, and it is highly likely that several more drugs will be used for repurposing drug indications in the future (Figure 2).

### 5.1. Methodology for Searching for New Repurposing Drugs

**Figure 3:**
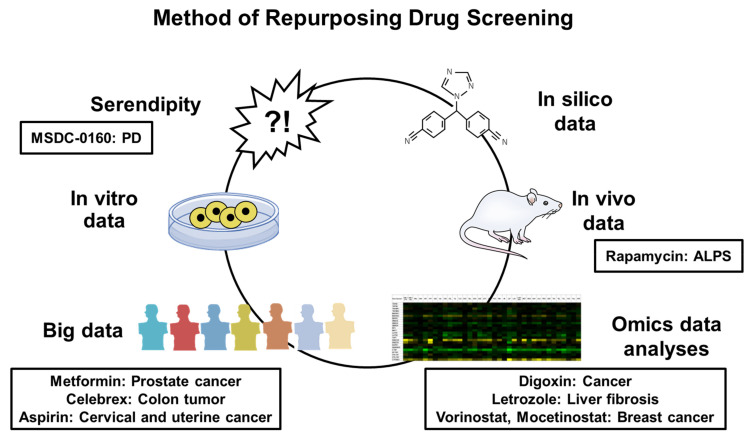
Methods of Repurposing Drug Screening. PD, Parkinson’s disease, ALPS, autoimmune lymphoproliferative syndrome.

As previously mentioned, repurposing drugs is a potential method for discovering new drug therapies with cost and time savings. The main methods for searching for new repurposing drugs are described below (Figure 3).

#### 5.1.1. Based on Clinical Big Data for Original Indication

First, there are methods for discovering therapeutic effects for other diseases based on clinical big data for the original indication. Although diabetes increases the incidence of various types of cancer [80,81], including colorectal cancer [82], gastric cancer [83], hepatocellular carcinoma [84], lung cancer [85], leukemia [86], meningioma [87], prostate cancer [52], ovarian cancer [88], and breast cancer [89], a 2006 report by Bowker et al. stated that cancer mortality rates vary depending on the type of diabetes treatment, with data from 10,309 diabetic patients showing that the metformin-using group had significantly lower cancer mortality rates than the sulfonylurea-using and exogenous insulin-using groups [90]. An example is the repurposing of the diabetes drug, metformin, for the treatment of various cancers, including prostate cancer. In detail, a Canadian cohort study including 3837 patients reported that metformin administration reduced both all-cause and prostate-cancer-specific mortality in patients with prostate cancer with diabetes mellitus [52]. The use of metformin has been suggested to be effective in other cancers as well; the MA32 study, including 2521 breast cancer patients, investigated whether 5-year metformin administration (versus placebo) improves invasive disease-free survival in early-stage breast cancer [91]. This method is frequently useful when the original indication has several patient populations, including diabetes. Moreover, safety and efficacy are often ensured as it is based on actual clinical data; however, this approach is less useful for rare and neglected diseases.

#### 5.1.2. In Silico Approach

In silico approach is a computer-based method that includes access to existing drug databases to search for new indications and machine learning, using similarities to useful disease structures and the pharmacophore of existing drugs as a reference. It is believed to be particularly useful for rare and neglected diseases [40]. First, in general, computational methods (in silico approach) are often used for drug development and the discovery of new drugs [92]. They are used for various applications including structure-based design of small-molecule drugs; putative ligand binding site prediction; steric structure and properties of macromolecule prediction, including enzymes and antibodies; antibody modeling; and undesirable pharmacokinetic property and potential toxicity evaluation. Examples of software used in research include BIOVIA Discovery Studio [93].

The computational approach can be used as a tool to complement experiments from the perspective of target- and ligand-cased strategies, including to identify transporter inhibitors (human peptide transporter 1 [hPEPT1] [94], P-glycoprotein (P-gp) [95], human organic cation/carnitine transporter (hOCTN2) [96,97], and human apical sodium-dependent bile acid transporter (ASBT)) [98]. The inhibition of these receptors may presumably be associated with rhabdomyolysis (hOTCN2), severe drug adverse reactions (ASBT), drug–drug interactions (hPEPT1 or P-gp), and colon cancer (ASBT).

The similarity of structures in 2D and 3D can be immediately used for drug repurposing. Furthermore, these attempts have been successful in the discovery of metabolite mimics in *Mycobacterium tuberculosis* [99] and in studies predicting drug–drug cross reactivity [100]. For example, Li Y Y et al. used a docking approach to narrow down 78 unique human drug targets from 1055 known drugs (from DrugBank) and noted 52 interactions of interest [101].

Moreover, machine learning has been put to practical use in the search for new drug repurposing, allowing tens of thousands of drug/protein interactions to be analyzed from public resources (e.g., DrugBank and SuperTarget).

#### 5.1.3. Comprehensive Analysis In Vitro or In Vivo

The next method is screening by in vitro or in vivo drug administration. Multiple existing drugs are administered to experimental systems in vitro or in vivo, and proteomics, transcriptomics, and comprehensive genetic analysis are often performed to search for new repurposing drugs [102]. For example, in breast cancer, new repurposing drugs (e.g., Vorinostat and Mocetinostat) are searched for on the basis of the results of genetic analysis with reference to large genetic databases, such as TCGA [44,76].

### 5.2. Usefulness of Repurposing Drugs in Immunotherapy

Immunotherapy is a therapeutic option which suppresses or enhances the patients’ immune system to treat autoimmune diseases and malignancy [1,2,3]. Due to the difficulty in developing new drugs and therapies in this field, there is an unmet need to discover the new therapeutic options in a cost- and time-effective manner. Therefore, in this study, we introduced repurposing drugs used as immunotherapy. For example, digoxin has been used for congestive heart failure and arrhythmia; however, it also affects HIF-1 molecules, which may act in immune response regulation and be newly indicated for prostate cancer treatment. The discovery of this new indication was triggered by a comprehensive genetic analysis experiment, wherein 3120 drugs were administered to cell lines in vitro [40]. In the following paragraphs, we will discuss the usefulness of the search for repurposing drugs using comprehensive genetic analysis.

### 5.3. Usefulness of Examining Changes in Gene Expression

#### 5.3.1. The Role of Examining Gene Expression in Searching for New Repurposing Drugs

In the search for repurposing drugs, exhaustive genetic analysis is useful as a method for detecting effective drugs for new target indications. As previously mentioned, the results of the exhaustive gene analysis for digoxin have led to the discovery of new indications for immunotherapy. Therefore, of the three previously mentioned exhaustive analyses, including proteomics, transcriptomics, and exhaustive genetic analysis, we will discuss exhaustive genetic analysis in detail.

#### 5.3.2. Cancer Drug Screening from the Perspective of Genetic Alteration

Attempts to develop cancer therapies on the basis of gene expression data have been made for various types of carcinomas. For example, studies based on gene expression data have been conducted in endometrial cancer [103], lung adenocarcinoma [104], and bladder cancer [105]. Regarding breast cancer, as we have introduced here, the search for repurposing drugs on the basis of gene expression data is underway. Specifically, candidate drugs for repurposing are selected on the basis of gene changes in breast cancer specimens downloaded from the TCGA database [106] using structural, gene change, and drug target network analysis.

#### 5.3.3. Discovery of New Repurposing Drugs Using Chimeric Mice Gene Expression Analysis

We performed a comprehensive genetic analysis, including liver fibrosis-related genes, by using human liver chimeric mice for the administration of existing drugs to search for new repurposing drugs. Here, the advantage of using human hepatocyte chimeric mice is that only hepatocytes are replaced by human ones in the livers of human hepatocyte chimeric mice [107]; therefore, the results of genetic analysis using human microarray can be interpreted as changes in hepatocytes rather than in nonparenchymal cells of the liver, including HSCs, Kupffer cells, and endothelial cells. The advantage of using human hepatocyte chimeric mice (PXB mice) is that predicting the changes that may occur in the human liver is possible, and it is particularly useful to observe hepatocyte-specific changes as well. As the result indicates, our report also showed a dose-dependent genetic change of letrozole in hepatocytes; however, no genetic change in HSCs was observed. Consequently, letrozole was observed as a new repurposing drug that suppresses liver fibrosis-related genes, including YAP-CTGF; the in vitro suppression of these genes was observed in a volume-dependent manner. In vivo experiments using two different liver fibrosis mouse models showed a weakening effect on liver fibrosis, and we reported that along with YAP-CTGF, HSD17B13, which is involved in retinoic acid metabolism [108,109] and nonalcoholic fatty liver disease [110], was associated with this effect [49].

## 6. Conclusions

The development of new drugs typically requires much investment and time; however, repurposing drugs is a method that has the potential for saving cost and time. Several methods are employed to search for repurposing drugs, including clinical big data in the original indication, computational structural analysis, existing drug databases, and drug screening by analyzing genetic changes in vitro and in vivo. The effective use of these methodologies would lead to the discovery of new drug treatments with minimal cost and time. Subsequently, it was suggested that the use of animal models, including human hepatocyte chimeric mice (PXB mice), would lead to a more accurate search for repurposing drugs.

## Figures and Tables

**Table 1:** Repurposing drugs for immunotherapy (approved or under trial).

No.	Drug Name	Original Indication	New Indication	Mechanism	Status	Method of Discovery	Refs
1	Rapamycin	Prevents organ transplant rejection	Autoimmune lymphoproliferative syndrome	Immunosuppression, decreases ds-DNA IgG	Approved	in vitro and in vivo	[14,15]
2	Metformin	Type 2 diabetes	Advanced prostate cancer	Inhibits the mTORC1 pathway, up-regulates AMPK	Phase II	Big data	[16,17,18]
3	Pentostatin	Chemotherapy for specific types of leukemia T-cell related	B-cell-related, called hairy cell leukemia	Immunosuppression, adenosine deaminase inhibitor	Approved	Clinical data	[19,20]
4	MSDC-0160	Type 2 diabetes	Parkinson’s disease	Inhibits inflammation and nerve death	Phase II completed	Pathological similarities (serendipity)	[21,22]
5	Aspirin	Pain and inflammatory disorders	Cervical and uterine cancer	Enhances PD-1 inhibition	Phase II	Big data	[23,24,25]
6	Celecoxib	Osteoarthritis	Colon tumor	Inhibits COX-2 receptors, NF-kB activity and interferes PPAR to DNA.	Approved	Big data	[26,27]
7	Niclosamide	Helminthic	Colorectal cancer	Inhibits PI3K/Akt, Wnt/β-catenin, JAK/STAT, NF-kB signaling, PD-L1 expression	Phase II	Gene expression analysis	[28,29,30,31,32]
8	Denosumab	Skeletal-related events in patient with solid tumors and multiple myeloma	Giant cell tumor of bone	Modulates RANK-RANKL signaling	Approved	In vitro and in vivo	[33,34]
9	Riluzole	Amyotrophic lateral sclerosis	Advanced solid tumor	Inhibits glutamate release	Phase I	In vitro and in vivo	[35,36,37,38,39]
10	Digoxin	Congestive heart failure and arrhythmia	Cancer	Inhibits Src and HIF-1	Phase I completed	In vitro and in vivo	[40,41,42]

ds-DNA, double-stranded-DNA, mTORC1, mammalian target of rapamycin complex 1, COX-2, cyclooxygenase-2, NF-kB, nuclear factor-kappa B, PPAR, peroxisome proliferator-activated receptor, PD-1, Programmed death receptor-1, PI3K, phosphatidylinositol 3′ -kinase, AKT, Protein Kinase B, JAK, Janus kinase, STAT, signal transducers and activator of transcription, PDL-1, Programmed death-ligand 1.

**Table 2:** Repurposing drugs screened by gene expression analyses.

No.	Drug Name	Original Indication	New Indication	Mechanism	Status	Method of Discovery	Refs
1	Vorinostat	Cutaneous T-cell lymphoma	HER2-positive breast cancer	HSP90 acetylation	Before clinical trials	Gene expression microarray	[43,44]
2	Mocetinostat (MGCD0103)	Hodgkin’s lymphoma	HER2-positive breast cancer	Induces USP-17	Before clinical trials	Gene expression microarray	[44,45,46]
3	Menadione	Vitamin K supplementation	Breast cancer	Affects F10 and EGFR genes and has an anti-proliferative action on breast cancer cells	Before clinical trials	Gene expression microarray	[46,47,48]
4	Letrozole	Chemotherapy for breast cancer	Liver fibrosis	Inhibits the YAP-CTGF pathway and regulation of retinoic acid metabolism	Before clinical trials	Gene expression microarray	[49]

HER2, human epidermal growth factor receptor 2, HSP90, Heat Shock Protein 90, USP, ubiquitin-specific protease, YAP, Yes-associated protein, CTGF, connective tissue growth factor.
